# Targeted Sequencing Identifies SNPs Associated with Antimalarial Drug Resistance and the CSP Vaccine Antigen in *Plasmodium falciparum* from Southwest Cameroon

**DOI:** 10.3390/ijms262110764

**Published:** 2025-11-05

**Authors:** Mary T. Efeti, Sandra N. Fankem, Mariama T. Diallo, Methodius S. Lahngong, Nelson L. Acha, Robert A. Shey, Kristiaan Demeyer, Jacob Souopgui, Stephen M. Ghogomu, Rose Njemini

**Affiliations:** 1 Frailty in Ageing Research Group, Vrije Universiteit Brussel, Laarbeeklaan 103, B-1090 Brussels, Belgium; mary.teke.efeti@vub.be; 2 Department of Gerontology, Faculty of Medicine and Pharmacy, Vrije Universiteit Brussel, Laarbeeklaan 103, B-1090 Brussels, Belgium; 3 Department of Biochemistry and Molecular Biology, Faculty of Science, University of Buea, Buea P.O. Box 63, Cameroon; lekeayinelson@gmail.com (N.L.A.); sheynce@gmail.com (R.A.S.); stephen.ghogomu@ubuea.cm (S.M.G.); 4 Laboratory of Embryology and Biotechnology, Department of Molecular Biology, Faculty of Science, Université Libre de Bruxelles, 6041 Gosselies, Belgium; sandra.fankem.noukimi@ulb.be (S.N.F.); mariama.telly.diallo@ulb.be (M.T.D.); jacob.souopgui@ulb.be (J.S.); 5 Laboratory of Pharmacognosy, Center for Interdisciplinary Research on Medicine (CIRM), University of Liege, 4000 Liege, Belgium; lahngongmethodius@gmail.com; 6 Laboratory of In Vitro Toxicology and Dermato-Cosmetology (IVTD), Department of Analytical, Applied Chemometrics and Molecular Modeling (FABI), Faculty of Medicine and Pharmacy, Vrije Universiteit Brussel, 1050 Ixelles, Belgium; kdemeyer@vub.be

**Keywords:** malaria, drug resistance, single-nucleotide polymorphisms, targeted sequencing, vaccine

## Abstract

Malaria is a major public health challenge in low- and middle-income countries with significant socio-economic impacts. While chemotherapy has greatly contributed to malaria control, the widespread emergence of resistance to antimalarial drugs threatens progress towards elimination goals. In parallel, the recent rollout of the RTS,S/AS01 and R21/Matrix-M malaria vaccine—targeting the *Plasmodium falciparum* circumsporozoite protein (CSP)—offers a new prevention tool but may be influenced by parasite genetic diversity. This study investigated the genetic architecture of *Plasmodium falciparum* circulating in a community in the Southwest Region of Cameroon. Seventy-two blood samples were analyzed using targeted Oxford Nanopore sequencing of *pfcrt*, *pfmdr1*, *pfdhfr*, *pfdhps*, *pfkelch13* and *pfcsp* genes. We observed a high prevalence of *pfdhfr* mutations (98.6% N51I, 98.6% C59R, 97.7% S108N) and *pfmdr1* Y184F (76.1%) mutation. Mutations in *pfdhps* (54.2% S436A, 2.8% A437G, 38.9% A581G) were also observed. No WHO-validated *pfkelch13* artemisinin resistance markers were found; however, K189T (63.4%) and R255K (4.2%) variants were detected. Nineteen non-synonymous SNPs were identified in *pfcsp*, reflecting natural background variations as vaccination status was not known. These findings support the continued use of artemisinin-based combination therapies and underscores the need for sustained molecular surveillance of both antimalarial drug resistance and vaccine-related polymorphisms, to inform malaria control strategies.

## 1. Introduction

Malaria is a life-threatening parasitic disease caused by *Plasmodium* species and transmitted by infected female *Anopheles* mosquitoes. Several *Plasmodium* species are known to infect humans including, *Plasmodium falciparum*, *Plasmodium vivax*, *Plasmodium malariae*, *Plasmodium ovale*, *Plasmodium knowlesi and Plasmodium simium* [1,2]. Despite continuous efforts to combat the disease, malaria remains a major public health challenge in low and middle-income countries [3,4], imposing long-term socio-economic burdens on affected populations [5]. According to the WHO 2024 Malaria Report, an estimated 263 million malaria cases and 723,000 deaths occurred in 2023 across 83 malaria-endemic countries, with the WHO African Region accounting for nearly 90% of the global burden [1]. *Plasmodium falciparum* (*P. falciparum*) remains the deadliest species in Africa [1,6], where it disproportionately affects children under five, causing 73.7% of global malaria deaths [1,6,7]. In Cameroon, malaria is responsible for about 50% of hospitalizations [8,9]. The country is among the 15 highest-burdened malaria countries, contributing about 2.8% of global malaria cases in 2023, making it the 12th most affected country worldwide [1].

Given the enormous disease burden, several strategies have been implemented to strengthen the control and elimination of malaria, including vector control, chemoprevention, and vaccine development. Recently, the WHO approved two malaria vaccines for broad use—RTS,S/AS01 in 2021 and R21/Matrix-M in 2023 [1,10,11]—both recommended for children (aged 5–17 and 5–36 months, respectively) in regions with moderate to high *P. falciparum* transmission [1,12,13]. Cameroon was the first country to introduce the RTS,S/AS01 vaccine into its Expanded Programme on Immunization (EPI), and by January 2024, the four-dose RTS,S/AS01 vaccine had been rolled out in 42 high-risk health districts across the 10 regions [14,15]. Both vaccines were developed using the C-terminal region (CTR) of the *P. falciparum* circumsporozoite protein (CSP) from strains 3D7 and NF54, respectively [16]. However, several single-nucleotide polymorphisms (SNPs), particularly within the CSP C-terminal region, have been reported among *P. falciparum* strains across diverse populations [17,18,19]. These SNPs may alter antigenicity, enable immune evasion, and potentially reduce vaccine effectiveness [20,21].

While the quest to develop a potent malaria vaccine continues, the use of antimalarial drugs has remained the cornerstone for malaria control and elimination over the past two decades. Quinine, the earliest widely used therapy introduced in the 17th century, was eventually replaced by chloroquine, considering its efficacy, affordability, and ease of use [22,23]. However, resistance emerged in the 1950s and 1960s, primarily due to mutations in the *pfcrt* (*Plasmodium falciparum* chloroquine resistance transporter) gene [23,24,25]. This led to the withdrawal of chloroquine as a frontline treatment [26,27]. A key example is the *pfcrt* K76T mutation, which spread globally, causing complete treatment failure with chloroquine and serving as a validated marker for chloroquine resistance [28,29]. To address rising resistance, new drugs were introduced, including sulfadoxine-pyrimethamine (SP, Fansidar) [22], which is still used in Cameroon for malaria chemoprophylaxis in children and pregnant women [30,31]. Unfortunately, mutations in the *pfdhfr* (dihydrofolate reductase) and *pfdhps* (dihydropteroate synthase) genes such as N51I, C59R, S108N (*pfdhfr*), and A437G, K540E (*pfdhps*) have been reported to reduce SP efficacy, leading to its restriction in many African countries [30,32,33,34,35]. To mitigate resistance, the WHO recommended a shift from monotherapy to artemisinin-based combination therapy (ACT). Artemisinin and its derivatives (artesunate, artemether, and dihydroartemisinin) are combined with partner drugs such as lumefantrine, amodiaquine, or piperaquine, to ensure rapid parasite clearance and delay resistance. ACT is now the WHO-recommended first-line treatment for most uncomplicated *P. falciparum* malaria cases [36]. In Cameroon, artesunate-amodiaquine (ASAQ) was approved as the first-line treatment of malaria in 2004, with artemether-lumefantrine (AL) introduced as an alternative ACT in 2006 [37].

Nevertheless, *P. falciparum* has developed resistance to artemisinin and its derivatives [36,38,39]. In 2010, artemisinin resistance was reported in Southeast Asia and was linked to mutations in the *kelch13* gene, which allows the parasite to enter a quiescent, prolonged ring-state [36]. Thirteen SNPs in the *pfk13* BTB/POZ and propeller domains are now classified as WHO-validated markers of partial artemisinin resistance [40], while several others remain under evaluation [22,38,39,41,42,43]. Outside these domains, the K189T and E252Q mutations have been frequently reported but not yet validated [40]. In addition, mutations in the *pfmdr1* gene—including N86Y, Y184F, S1034C, N1042D, and D1246Y—have been associated with resistance and altered drug responses [44,45]. The emergence and spread of resistant *P. falciparum* genotypes is a growing global concern, with increasing evidence reported across Africa [38,41,42,46,47,48]. Resistance to ACTs poses a major threat to the goals of the WHO Global Technical Strategy for Malaria (GTS), which aims to reduce malaria incidence and mortality by at least 90% by 2030. Molecular markers of resistance therefore serve as essential tools for the surveillance and monitoring of antimalarial drug effectiveness across endemic regions in Asia and Africa. In general, the greater the number of resistant-associated biomarkers detected in a locality, the higher the likelihood that ACT- and other antimalarial-resistant parasites are circulating. As strongly recommended by the WHO [6], endemic countries must continuously monitor the spread of these resistance markers to guide timely updates of national treatment policies. This is particularly important given that current vaccines provide only partial protection and are mainly limited to children, and no alternative drug has yet replaced ACTs as the cornerstone for treatment. In this study, we investigated the genetic architecture of the *P. falciparum* from infected individuals in the Southwest Region of Cameroon, where recurrent malaria episodes persist despite full treatment courses and is amongst the 10 regions of the RTS,S/AS01 vaccine roll-out. Using targeted sequencing, we identified mutations in *pfcrt*, *pfmdr1*, *pfdhfr*, *pfdhps*, and *pfkelch13* genes associated with antimalarial drug resistance, as well as multiple polymorphisms in the C-terminal region of *pfcsp* that may influence the effectiveness of malaria vaccines in this locality.

## 2. Results

### 2.1. General Characteristics of Study Population

This was a cross-sectional, hospital-based study carried out in the Southwest Region of Cameroon from March to June 2024. A total of 460 individuals with a medical doctor’s request for malaria testing were enrolled, of whom 171 (37.2%) tested positive for *Plasmodium* using the malaria Rapid Diagnostic Test (RDT). Among the RDT-positive cases, 40.9% (70/171) were confirmed by Giemsa-stained microscopy as *P. falciparum*, with a minimum of 106 parasites per 200 white blood cells. Most of the malaria-positive patients were female (59.1%, 101/171), while 40.9% (70/171) were male. Children under five years accounted for 19.3% (33/171) of cases, while the largest group was young adults aged 25-64 years (29.3%, 50/171). and 6.4% (11/171) of participants were elderly (>65 years). About 61% (94/154) presented with fever (temperatures >37.8 °C) [Table 1]. Information on the malaria vaccination status of the child participants was not recorded at the time of the study. In total, 170 samples were available for downstream molecular analysis.

### 2.2. Screening of Samples and Polymerase Chain Reaction (PCR) Amplification of pfkelch13, pfdhfr, pfdhps, pfcrt, pfmdr1, and pfcsp Gene Targets

**Figure 1:**
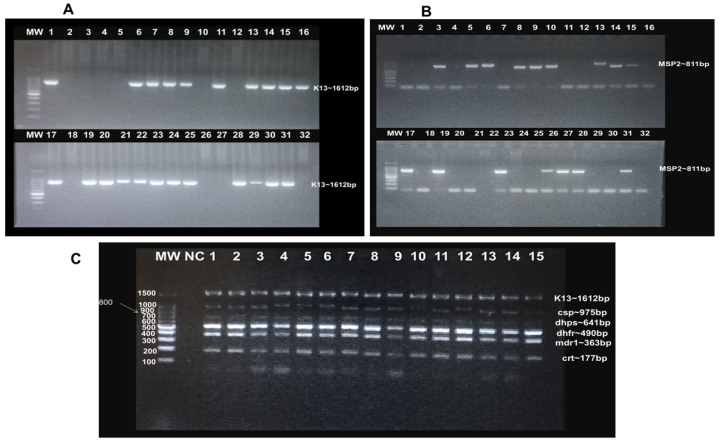
Agarose gel electrophoresis confirmation of *P. falciparum* active infection and targeted PCR amplification: (**A**): Screening by *kelch13* amplification: Lanes 1, 6, 7, 8, 9, 11, 13, 14, 15, 16, 17, 19, 20, 21, 22, 23, 24, 25, 28, 29, 30, 31 = positive samples, while lanes 2, 3, 4, 5, 10, 12, 18, 26, 27, 32 = negative samples. (**B**): Screening by *msp2* amplification: Lanes 3, 5, 6, 8, 9, 9, 10, 13, 14, 17, 19, 23, 26, 27, 28, 31 = positive samples, while lanes 1, 2, 4, 7, 11, 12, 16, 18, 20, 21, 22, 24, 25, 29, 30, 32 = negative samples. (**C**): PCR amplification of *P. falciparum* antimalarial drug-resistance and vaccine gene targets: Bands correspond to *kelch13*, *pfcsp*, *pfdhps*, *pfdhfr*, *pfmdr1* and *pfcrt* amplicons, respectively. Lane 1–15 = clinical samples. MW = 100 bp DNA ladder; NC = negative control.

DNA extraction was performed using the Zymo Quick-DNA^TM^ Miniprep Plus Kit following the manufacturer’s protocol. The 170 RDT malaria positive samples were screened by PCR amplification of the *P. falciparum* merozoite surface protein 2 (*msp2*) and *kelch13* genes (Figure 1A,B). Of these samples, 68.88% (117/170) were confirmed to have an active parasite infection. Subsequently, multiplexed PCR was performed to amplify five *P. falciparum* genes associated with antimalarial drug resistance, along with the malaria vaccine target gene. These included *kelch13*, multidrug-resistant protein 1 (*pfmdr1*), dihydrofolate reductase (*pfdhfr*), dihydropteroate synthase (*pfdhps*), chloroquine resistance transporter (*pfcrt*), and the circumsporozoite protein (*pfcsp*) gene, which is the target of the current malaria vaccine. A 2% agarose gel electrophoresis was performed to verify amplicon sizes and integrity (Figure 1C).

### 2.3. Targeted Sequencing Identified Single-Nucleotide Polymorphisms in Gene Targets

**Figure A1:**
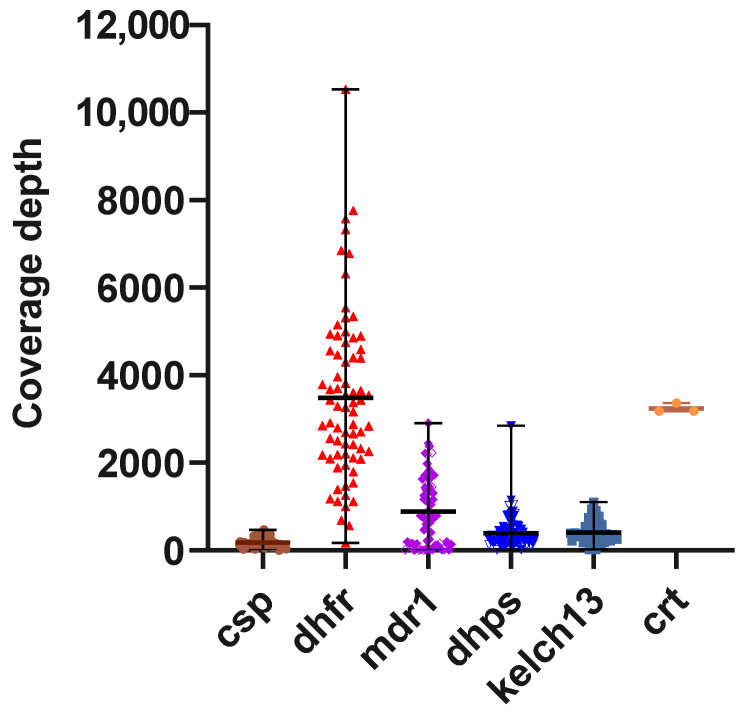
Coverage-depth for Amplicon targets: The y-axis shows the median number of reads covering each amplicon target per sample. The average coverage-depth was 178.0, 3482, 886.7, 396.3, 407.7 and 2850.7 for *pfcsp*, *pfdhfr*, *pfmdr1*, *pfdhps*, *pfkelch13* and *pfcrt* amplicon targets, respectively.

The six gene targets were successfully amplified in 72 samples that were positive for both *msp2* and *kelch13*. Amplicons were purified and quantified yielding a minimum concentration of 99.96 ng/µL and an A260/280 ratio of 1.69 (Table 2) as determined by Qubit fluorometric quantification (dsDNA high sensitivity, Promega). All 72 samples were therefore included in targeted sequencing using the Oxford Nanopore Technologies (ONT) platform. Nanopore sequencing was performed in two multiplexed batches using an ONT Flongle R10.1 flow cell. In the first batch of 40 samples, a basecalling-yield of 100% (189.57 Mb/166.28 Mb) was obtained in 7 h with 345.11 K reads generated. For the second batch of 32 samples (including 8 duplicates for internal quality control), a basecalling-yield of 96.05% (274 Mb/294.42 Mb) was obtained in 20 h with 613.98 K reads generated (Table 3). The generated reads were trimmed, filtered, and aligned to the *P. falciparum* 3D7 reference genome using Minimap2, and variant calling was performed with Clair3. The average coverage-depth was 178.0, 3482, 886.7, 396.3, 407.7 and 2850.7 for *pfcsp*, *pfdhfr*, *pfmdr1*, *pfdhps*, *pfkelch13* and *pfcrt* amplicon targets, respectively (Appendix B, Figure A1). A total of 98 variants were identified: 81 SNPs, 4 insertions, and 13 deletions (Appendix A, Table A2).

### 2.4. Frequency of Identified Single-Nucleotide Polymorphisms Associated with Drug Resistance

Analysis of antimalarial drug-resistance genes identified a total of 11 non-synonymous mutations in the study population: two in the *pfkelch13* gene (K189T and R255K), one in the *pfcrt* gene (K76T), three in *pfdhfr* (N51I, C59R and S108N), four in *pfdhfps* (S436A, A437G, A581G and A613S) and one in *pfmdr1* (Y184F) (Table 4). The prevalence of the K189T and R255K was 63.4% (45/71) and 4.1% (3/71), respectively, corresponding to a wild-type allele prevalences of 36.6% and 95.8%, respectively. A high prevalence of the *pfdhfr* point mutations associated with pyrimethamine resistance was observed: 98.6% (70/72) for N51I, 98.6% (70/72) for C59R, and 97.8% (69/72) for S108N. The *pfdhfr* triple mutant (N51I/C59R/S108N) was present in almost all samples, with a prevalence of 98.6% (71/72). For the *pfdhps* point mutations associated with sulfadoxine resistance, the prevalence was 54.2% (39/72) for S436A, 2.8% (2/72) for A437G, 28.9% (28/72) for A581G, and 44.4% (32/72) for A613S. The combined *pfdhfr + pfdhps* quadruple mutation (N51I/C59R/S108N + A437G), associated with SP resistance, was detected in 2.8% (2/72) of samples. The *pfmdr1* Y184F mutation, reported to be associated with resistance to amodiaquine, mefloquine, lumefantrine, and possibly artemisinin, was found in 75% (54/72) of the samples (Table 4). Finally, the *pfcrt* K76T mutation, which is associated with chloroquine resistance, was identified in only 4.2% (3/72) of the samples.

### 2.5. Frequency of SNPs in the P. falciparum Circumsporozoite Protein That May Impact Malaria Vaccine Efficacy

**Figure 2:**
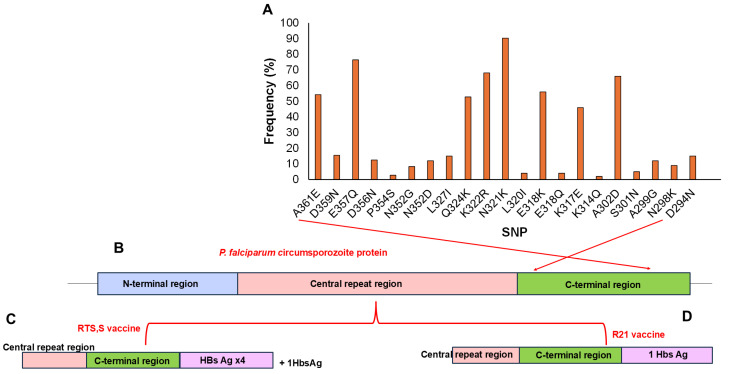
Representation of identified SNPs in the *P. falciparum* circumsporozoite protein (CSP) vaccine target: (**A**) Frequency of SNPs identified within amino acid positions 268–361 (x-axis, right to left) corresponding to the C-terminal region of the *pfcsp* segment in the *Pf*3D7 reference strain. Structure of the pfcsp protein (**B**), Structure of the RTS,S vaccine target (**C**), and Structure of the R21 vaccine target (**D**).

In this study, a total of 51 *pfcsp* SNPs were identified in the population with 37.25% (19/51) being non-synonymous and 62.27% (32/51) synonymous mutations. The non-synonymous mutations were observed in amino acid positions spanning 268–361 (Figure 2A) which corresponds to the C-terminal region of the circumsporozoite protein (273–397) of the *pf*3D7 reference strain (Figure 2B). Several non-synonymous SNPs occurred in the Th2R segment of the C-terminal region at high frequencies (>5%), including L320I (5.6%), K317E (91.7%), E318K (77.8%), N321K (90.3%), K322R (68.1%), Q324K (52.8%) and L327I (20.8%). Other mutations such as K314Q (2.8%), were detected but at lower frequencies (<5%). In the Th3R segment, non-synonymous SNPs identified at high frequency (>5%) included N352D/G (12.5/8.3%), D356N (12.5%), D357N (15.5%), D359Q (15.6%), and A361E (54.2%). Additional mutations such as P354S (2.8%) were present but at low frequencies (<5%). Other SNPs in the CTR identified at high frequencies (>5%) included N298K (12.5%), S301N (6.9%) and A302D (91.7%). The direct effects of these SNPs on vaccine efficacy were not assessed in this study.

## 3. Discussion

Continuous monitoring of the spread of molecular markers of drug resistance in malaria-endemic countries is critical as this not only improves treatment options for patients with recurrent infections but also guides national treatment, control, and prevention strategies. This study assessed the genetic architecture of *P. falciparum* circulating in a community in the Southwest Region of Cameroon, where recurrent malaria episodes have been reported despite completion of recommended treatment regimens; this region is amongst the 10 regions included in the RTS,S/AS01 vaccine roll-out. Here, we sequenced the antimalarial drug-resistance gene targets (*pfcrt*, *pfdhfr*, *pfdhps*, *pfkelch13*, *pfmdr1*), as well as the vaccine target (*pfcsp*), in 72 actively infected samples. Nanopore sequencing identified a total of 11 non-synonymous SNPs in the population: two in the *pfkelch13* gene (K189T and R255K), one in the *pfcrt* gene (K76T), three in *pfdhfr* (N51I, C59R, and S108N), four in *pfdhfps* (S436A, A437G, A581G, and A613S), and one in *pfmdr1* (Y184F) (Table 4). Although no *kelch13* candidate mutations validated by WHO for artemisinin resistance were identified, the K189T 63.4% (46/72) and, for the first time in this region, R255K 4.2% (3/72) variants were detected. These mutations have been reported in several studies examining genetic variations associated with drug resistance. Both K189T and R255K have been linked to delayed parasite clearance in some *P. falciparum* isolates in Southeast Asia and Africa [52,53,54] but their direct role in mediating resistance remains inconclusive. The *pfcrt* K76T mutation, detected in only 4.2% (3/72) of samples, is a well- established marker of chloroquine resistance and can also contribute to cross-resistance with other antimalarials, such as amodiaquine and quinine. This reduces parasite sensitivity to multiple drugs and complicates treatment efforts [28,29]. Chloroquine resistance, which first emerged in the Southwest Region of Cameroon [55,56], spread nationwide and led to the replacement of chloroquine as the first-line therapy with alternatives such as amodiaquine, sulfadoxine–pyrimethamine (SP), and, later, artemisinin-based combinations including artesunate–amodiaquine (ASAQ) and the artemether–lumefantrine (AL) in response to rising drug resistance [30,31,37]. While some studies have suggested reconsidering chloroquine because of an apparent resurgence of sensitive strains [57,58,59], our findings do not clearly indicate such a trend, partly due to incomplete *pfcrt* genotype information for some isolates. The *pfmdr1* Y184F mutation detected in most of the samples (75%, 54/72) is noteworthy. Together with the *pfcrt* K76T genotype, it has been frequently reported across endemic regions and is under investigation for its potential role in modulating parasite susceptibility to ACT partner drugs [44,45]. Continued molecular surveillance remains essential to monitor these alleles and their possible clinical relevance.

In Cameroon, SP is primarily used for intermittent preventive treatment of malaria in pregnancy (IPTp), intermittent preventive treatment of infants (IPTi), and seasonal malaria chemoprevention (SMC). Mutations in *pfdhfr* and *pfdhps* are responsible for SP resistance. In this study, we observed very high prevalence of *pfdhfr* mutations [98.6% (71/72) N51I, 98.6% (71/72) C59R, and 97.7% (70/72) S108N] alongside *pfdhps* mutations [S436A (54.2%, 39/72), A437G (2.8%, 2/72), A581G (38.9%, 28/72) and A613S (44.4%, 32/71)]. These high frequencies are consistent with previous reports from several regions of Africa, where similar mutation patterns have been observed in association with reduced SP efficacy [30,32,50,51,60]. Although our study did not directly access drug efficacy, the high prevalence of these mutations suggests continued selection pressure that may favor the persistence and accumulation of resistant-*pfdhfr* and *pfdhps* alleles in this local parasite population. Importantly, the combined *pfdhfr + pfdhps* quadruple mutant genotype (51I/59R/108N + A437G), which was identified in only 2.8% of the isolates, has been linked in other studies to high-level SP resistance [30,49], raising concerns about the continued efficacy of SP in preventive programs.

The C-terminal region of the *P. falciparum* parasite circumsporozoite protein, from which the RTS,S and R21 malaria vaccines were developed, has been reported to exhibit several SNPs across different populations [17,18,19,21]. Some of these SNPs have been predicted in some studies [20,21] to affect antigenicity, potentially influencing vaccine effectiveness. Cameroon was the first country to introduce the malaria vaccine into its EPI in 2021. By 2024, the four-dose RTS,S/AS01 vaccine had been rolled out in 42 high-risk health districts across the 10 regions, including the southwest region [15,16]. This widespread rollout reflects Cameroon’s diverse geo-ecological profile and provides a unique opportunity to assess *pfcsp*’s genetic architecture and how this might affect vaccine effectiveness, particularly as vaccination expands across the country. In this study, a total of 51 *pfcsp* SNPs were identified, with 37.25% (19/51) being non-synonymous and 62.27% (32/51) synonymous mutations. Because the vaccination status of the child participants was not recorded in this study, the *pfcsp* SNPs reported herein are interpreted as representing the natural background diversity within circulating *P. falciparum* populations rather than any vaccine-driven adaptations. Several of these non-synonymous SNPs occurred in the Th2R segment of the CTR at a high frequency (>5%), including L320I (5.6%), K317E (91.7%), E318K (77.8%), N321K (90.3%), K322R (68.1%), Q324K (52.8%), and L327I (20.8%). The Th2R segment is recognized by CD4+ T-helper cells after antigen presentation and promotes B cell proliferation, leading to antibody production against CSP antigens during immunization [61,62]. Similarly, non-synonymous SNPs occurring in the Th3R segment of the CTR at high frequency (>5%) included N352D/G (12.5/8.3%), D356N (12.5%), D357N (15.5%), D359Q (15.55), and A361E (54.2%). The Th3R segment also stimulates CD4+ T-helper responses and indirectly activates CD8+ T-cell after vaccination with the CSP antigens [61,62]. This observation is consistent with previous studies reported by Maima et al., (2024), Amegashie et al., (2020), and Dieng et al., (2023) [17,20,21]. Though the direct effects of these SNPs on vaccine efficacy were not assessed in this study, they overlap with the SNPs previously reported in the CTR by Amegashie et al., (2020) [17]. Given that ThR2 and ThR3 regions are involved in the CD4+ and, to a lesser extent, CD8+ T cell responses, the presence of polymorphisms may help the parasite to evade host immune pressure, thereby compromising overall vaccine efficacy [20]. Our findings thus provide a valuable baseline for future monitoring of the potential selection of variants under increasing vaccine pressure. Hence, future studies integrating genomic surveillance with vaccination records and immune responses will better highlight the relevance of CSP polymorphisms to ongoing malaria vaccine rollout in Cameroon.

Given that our study sampled a single community in the southwest region, the relatively small sample size may limit the representativeness of our findings. We therefore recommend that future studies expand sampling to include larger populations and multiple districts across the southwest region, as well as other geo-ecological zones of Cameroon. This would provide representative and population-level data for both antimalarial drug-resistance and malaria CSP vaccine polymorphism surveillance. For *pfcsp*-vaccine related surveillance, we recommend future monitoring of the NANP-repeat and C-terminal regions, haplotype analysis, and antigenicity predictions.

## 4. Materials and Methods

### 4.1. Study Design and Ethical Considerations

This was a cross-sectional, hospital-based study conducted in the Southwest Region of Cameroon. The study was approved by the Cameroon National Ethical Committee for Research in Human Health (approval number: 2023/10/1602/CE/CNERSH/SP). Informed consent/assent forms were provided and thoroughly explained to all participants, before enrollment. All participants signed the consent/assent forms before enrollment. Participation was entirely voluntary, and participants were free to withdraw at any time. Confidentiality was strictly maintained throughout the data collection, analysis, and reporting. Consenting participants of all age groups confirmed positive for malaria by both RDT and microscopy were recruited after providing demographic, phenotypic, and laboratory information through a participant questionnaire.

### 4.2. Study Site and Sample Collection

The study sampled *P. falciparum* isolates from populations in the Southwest Region of Cameroon, particularly within the Muea Health District. This district lies within the equatorial forest zone of Southern Cameroon and is classified as holoendemic, with intense and perennial malaria transmission [49,63]. Samples were collected between March and June 2024, during the rainy season. Consenting participants with a medical doctor’s request for a malaria test, and who voluntarily completed the research questionnaire, were recruited. Two milliliters (2 mL) of venous blood were collected from each consenting participant under sterile conditions into labeled EDTA tubes by a laboratory technician. Samples were diluted in DNA/RNA Shield (in a ratio of 1:3) and stored at −20 °C for downstream molecular analysis. This procedure ensured the integrity of nucleic acids and inactivated potential pathogens during collection, storage, and transportation.

### 4.3. Malaria Parasitemia Determination

During sample collection, participants were prescreened with a malaria RDT, using 50 μL of whole blood (CareStart^TM^ Malaria (HRP2) Ag RDT, ACCESSBIO, Somerset, NJ, USA). Two drops of blood were then placed on a slide and air-dried for further analysis by light microscopy. Giemsa-stained thick and thin smears of peripheral blood were prepared (for some samples). The level of parasitemia was determined by counting the number of parasites per 200 white blood cells, assuming a standard white blood cell count of 8000/µL of blood [64]. A smear was considered negative only if no malaria parasites were seen in 100× high-power fields.

### 4.4. DNA Extraction and Purification

Genomic DNA was extracted from whole blood samples using the Zymo Quick-DNA^TM^ Miniprep Plus Kit (Zymo Research, Tustin, CA, USA). Briefly, diluted whole blood samples in DNA/RNA shield were thawed at room temperature (25 °C), and 20 µL of Proteinase K was added to 400 µL of the blood sample in a 1.5mL micro centrifugation tube. Mixtures were vortexed thoroughly and incubated at room temperature (25 °C) for 20 min. One volume (420 µL) of Genomic Binding Buffer was added, and the mixture was vortexed and transferred into a Zymo-Spin^TM^ IIC-XLR column (Zymo Research, Tustin, CA, USA) in a collection tube for centrifugation at 13,000× *g* for 1 min. The eluate was discarded, and 400 µL of DNA Pre-Wash Buffer was added to the spin column in a new collection tube and centrifuged at 13,000× *g* for 1 min. After discarding the flow-through, 700 µL of g-DNA Wash Buffer was added and centrifuged at 13,000× *g* for 1 min, followed by a second wash with 200 µL of g-DNA Wash Buffer under the same conditions but for 3 min to ensure removal of residual wash buffer. Thereafter, the spin column was transferred to a labeled 1.5 µL microcentrifuge tube, 30 µL of DNA Elution Buffer was added and the column was incubated at room temperature for 30 min. Columns were centrifuged at 14,000× *g* for 1 min, to elute DNA. The eluted DNA was stored at −20 °C for downstream analysis.

### 4.5. Molecular Identification of Actively Infected Samples and Amplification of Drug-Resistance and Vaccine Gene Targets

RDT-positive samples were further screened by PCR amplification of the *P. falciparum* merozoite surface protein 2 (*msp2*) and *kelch13* genes. Targeted PCR amplification was also performed for the *kelch13*, *crt*, *mdr1*, *dhfr*, *dhps*, and *csp* genes. Previously designed pairs of gene-specific primers (Appendix A, Table A1) were used, and PCR was performed using a 2720 Thermal cycler (Thermo Fisher Scientific, Foster City, CA, USA). PCR was performed in 100 μL PCR tubes. A total of 2 μL of each primer pair (10μM for *kelch13*, *mdr1*, and *crt* gene targets, and 50 μM for *dhfr* and *dhps*) in a final volume of 15 μL, containing 2 μL genomic DNA, 7.5 μL of PCR master mix, and 3.5 μL of distilled water. The PCR master mix contained standard reaction components (1× Taq buffer, 0.05 U/μL Taq polymerase, 2.0 mmol/L MgCl2, 0.4 mmol/L dNTPs, and 10× DNA loading dye). PCR cycling conditions followed primer-specific requirements, as described in Appendix A, Table A1.

### 4.6. Confirmation of PCR Amplicons by Agarose Gel Electrophoresis

A 2% agarose gel was prepared to analyze the screening and amplification products of gene targets in infected samples. Two grams of agarose were dissolved in 100 mL of 0.5× TAE (Tris Acetate EDTA) buffer with 1 µL/100 mL of SYBR Safe-DNA Gel Stain. The mixture was swirled and allowed to solidify. Five microliters of PCR product were loaded into each well, electrophoresed at 100 V for 45 min, and visualized under ultraviolet light using a Gel Doc XR+ (BioRad, Hercules, CA, USA).

### 4.7. Purification and Quantification of Amplicons

Individual targeted amplicons were pooled together and purified using the Wizard SV Gel and PCR Clean-Up System according to the manufacturer’s protocol.

The concentration of each purified pooled amplicon was determined using a NanoDrop spectrophotometer (EzDrop 1000 UV/Vis spectrometer, New Taipei City, Taiwan (R.O.C)). The spectrophotometer was blanked using 1 µL of nuclease-free water, after which 1 µL of PCR sample was loaded, the lid closed, and the concentration and purity measured at 260/280 nm absorbance (purity range 1.67–2.00). This was repeated for each sample. In parallel, DNA quantification was also performed using the Qubit fluorometer with a high-sensitivity dsDNA assay kit (Promega, Madison, WI, USA), to ensure accurate quantification for downstream library preparation.

### 4.8. Targeted Sequencing to Identify Single-Nucleotide Polymorphisms

**Figure 3:**
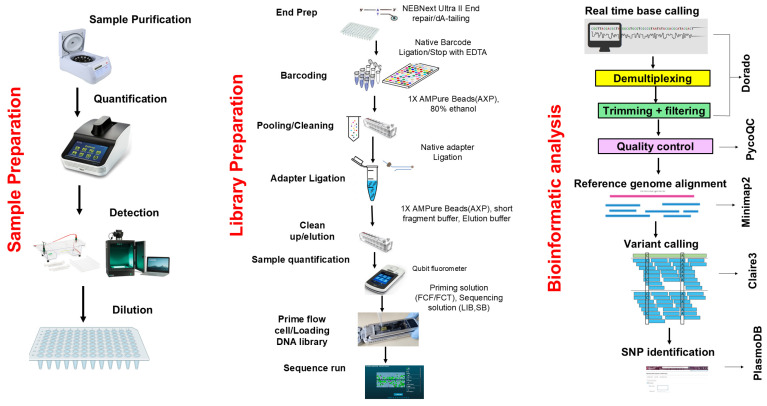
Overview of sequencing workflow.

For each clinical sample (PCR product), targeted sequencing was performed using the Oxford Nanopore Technologies (ONT) platform. The procedure encompassed library preparation, sequencing and bioinformatics analysis (Figure 3). A multiplex library was prepared using the ONT Ligation sequencing amplicon-Native Barcoding Kit 96 V14 (SQK-NBD114.96, Oxford, UK) according to the manufacturer’s instructions. Sequencing runs were performed on a Flongle R10.1 flow cell in two batches: the first with 40 clinical samples and a second with 32 clinical samples (including 8 duplicates for internal quality assessment). Real-time basecalling, demultiplexing and barcode trimming were performed using MinKNOW software version 24.06.16 (Bream 8.0.13, configuration 6.0.19, Dorado 7.4.14, MinKNOW Core 6.0.15) and the resulting raw data were generated in FASTQ files for downstream analysis.

### 4.9. Bioinformatics Analysis

Following real-time basecalling, demultiplexing, and barcode trimming, the FASTQ files were quality-checked using PycoQC tool version 2.5.2 [65]. Thereafter, the sequences were aligned to the *P. falciparum* 3D7 reference genome using Minimap2 version 2.28 [66] with the map-ont preset. SAMtools version 2.21 utilities [67] were used to sort and index the resultant alignments, generating sorted BAM files. In addition, coverage-depth statistics of the 3D7-aligned BAM files were determined using the same tool. Variant calling for SNPs identification was performed using Clair3 version 1.0.9 [68]. High-accuracy variant-calls with potential SNPs and Haplotype differences were extracted as VCF files. The SNPs were filtered by quality (score > 10) and coverage-depth (>5) using BCFtools version 1.21 [67]. Unique PlasmoDB IDs for each identified SNP were generated in R, and these IDs were queried against the PlasmoDB database (https://plasmodb.org/plasmo/app accessed on 25 February 2025) to determine whether the SNP identified were present in the database or were novel. The identified SNPs were downloaded into an Excel file for downstream statistical inference and interpretation.

### 4.10. Data Analysis

All data were entered and processed using Microsoft Excel version 2507. Data are expressed as means ± SD for continuous variables and as numbers (n) or percentages (%) for categorical variables.

## 5. Conclusions

In this study, we identified high prevalences of mutations in *pfdhfr* and others in *pfdhps* genes associated with sulfadoxine-pyrimethamine resistance. The high frequency of *pfmdr1* Y184F (75%) marker observed further suggests ongoing selection by antimalarial drug use in the population. No WHO-validated marker for artemisinin resistance was detected; however, the *pfkelch13* K189T and R255K mutations, were observed. Nineteen non-synonymous SNPs were also identified in the *pfcsp* gene likely reflecting natural background variations as vaccination status was not recorded; however, the potential impact of these SNPs on parasite recognition by vaccine-induced responses remains to be determined. Overall, these findings support the continued use of ACTs in this region but underscore the urgent need for sustained molecular surveillance of antimalarial drug-resistant markers, particularly of SP-resistant markers, to inform and strengthen malaria control strategies. Additionally, monitoring *pfcsp* polymorphisms alongside vaccination records in future studies will be important to evaluate potential impact on vaccine efficacy to inform regional control strategies.

## Figures and Tables

**Table 1:** General characteristics of study participants.

Characteristics		Percentages (*n*/N)
Gender	Male	40.9% (70/171)
	Female	59.1% (101/171
Age (Years)	<5	19.3% (33/171)
	5–14	20.5% (35/171)
	15–24	24.6% (42/171)
	25–64	29.2% (50/171)
	≥65	6.4% (11/171)
Temperatures (°C)	36.4–37.8	39% (60/154)
	≥37.8	61% (94/154)

**Table 2:** Concentration and absorbance of purified amplicons.

	N	Minimum	Maximum	Mean	Std. Deviation
DNA Conc (ng/ uL)	72	99.950	247.430	166.765	36.327766
A260/280	72	1.69	1.94	1.8538	0.05345
Valid N	72				

(N = Total samples included for targeted sequencing).

**Table 3:** Run Summary.

Sequencing Batch Code	ONT Kit	Number of Clinical Samples per Run	Run Time	Reads Generated	Estimated Bases	Basecalled Bases	Basecalled %
A	Flongle R10.1	40	7 h	345.11 K	166.28 Mb	189.57 Mb	100%
B	Flongle R10.1	32 (+8 duplicates)	19 h 52 min	613.98 K	294.42 Mb	274 Mb	96.05%
Total		72					

**Table 4:** Single-nucleotide polymorphisms associated with antimalarial drug resistance in the study population.

Gene Name	Associated Antimalarial Drug	Identified SNP	Allele Frequency %(*n*/N)	Status and Reference
Chloroquine-resistant transporter, *pfcrt*	Chloroquine	K76T	4.2% (3/72)	Resistant mutation [25,29]
Dihydrofolate reductase, *pfdhfr*	Pyrimethamine	N51I	98.6% (71/72)	Associated with SP resistance [28,33,49,50,51]
		C59R	98.6% (71/72)	
		S108N	97.7% (70/72)	
Dihydropteroate synthase, *pfdhps*	Sulfadoxine	S436A	54.2% (39/72)	Associated with SP resistance [28,33,49,50,51]
		A437G	2.8% (2/72)	
		A581G	28.9% (28/72)	
		A613S	63.4% (32/72)	
Multidrug resistance protein 1, *pfmdr1*	Several antimalarials	Y184F	75% (54/72)	Associated with mefloquine, lumefantrine, chloroquine and artemisinin resistance [44,45,51]
*Pfkelch13*	Artemisinin	K189T	63.4% (46/72)	May reduce susceptibility to artemisinin [52,53,54]
		R255K	4.2% (3/72)	
*Pfdhfr* triple mutant		N51I/C59R/S108N	98.6% (71/72)	SP resistance [28,33,50]
*Pfdhfr* + *Pfdhps* quadruple mutant		N51I/C59R/S108N+ A437G	2.8% (2/72)	Associated with SP resistance [33,50]

(*n*/N = number of samples with SNP/total samples).

## Data Availability

The raw FASTQ data is being submitted to NCBI. Also, the raw data supporting the conclusion of this article will be made available by the authors upon request.

## Abbreviations

The following abbreviations are used in this manuscript:

SNP	Single-nucleotide polymorphism
ACT	Artemisinin-based combination therapy
*pfcrt*	*Plasmodium falciparum* chloroquine resistant transporter
*pfdhfr*	*Plasmodium falciparum* dihydrofolate reductase
*pfdhps*	*Plasmodium falciparum* dihydropteroate synthase
*pfmdr1*	*Plasmodium falciparum* multidrug resistance 1
*pfcsp*	*Plasmodium falciparum* circumsporozoite protein
PCR	Polymerase chain reaction
ONT	Oxford Nanopore technology
SP	Sulfadoxine-pyrimethamine
EPI	Expanded Programme on Immunization
AL	Artemether-lumefantrine
ASAQ	Artesunate-amodiaquine
BTB/POZ	Bric-a-brac, Tramtrack, and Broad-complex/Poxvirus and zinc finger
WHO	World Health Organization
CTR	C-terminal region
GTS	Global Technical Strategy
RDT	Rapid Diagnostic Test
RTS,S/AS01	Repeat T epitopes and Surface antigen
IPTp	Intermittent preventive treatment of malaria in pregnancy
IPTi	Intermittent preventive treatment of infants
SMC	Seasonal malaria chemoprevention

## Appendix A

### Appendix A.1

**Table A1:** Thermocycling parameters for amplification of gene targets.

Gene Target	Primer Sequence (Forward and Reverse)	Reaction Conditions	Reaction Components
MSP2	5′-ATG AAG GTA ATT AAA ACA TTG TCT ATT-3′ 5′-ATA TGG CAA AAG ATA AAA CAA GTG TTG-3′	94 °C 3 min, 94 °C 30 s, 55 °C 1 min, 72 °C 1 min, 72 °C 4 min, 4 °C ∞, 40×	Go taq (7.5 µL), F/R primer (2 µL), _nf_H_2_O (3.5 µL), gDNA (3 µL), Total volume = 15 µL
*kelch13* ampl	5′-GGG AAT CTG GTG GTA ACA GC-3′ 5′-CGG AGT GAC CAA ATC TGG GA-3′	94 °C 3 min; 94 °C 30 s; 61 °C 1 min 30 s; 72 °C 1 min 30 s; 72 °C 10 min, 4 °C ∞; 40×	
*Kelch13* nested	5′-AAG CCT TGT TGA AAG AAG CA-3′ 5′-GGG AAC TAA TAA AGA TGG GC-3′		
*Pfcsp*	5′-TGG GAA ACA GGA AAA TTG GTA T-3′ 5′-TAC GAC ATT AAA CAC ACT GGA A-3′	94 °C 5 min; 94 °C 30 s; 58 °C 1 min; 72 °C 1 min 30 s; 72 °C 5 min, 4 °C ∞; 40×	Go taq (12.5 µL), F/R primer (csp 2 µL; dhfr 1.2 µL; dhps2 µL), _nf_H_2_O (3.3 µL), gDNA (4 µL), Total volume = 25 µL
*Pfdhfr*	5′-GTT TTC GAT ATT TAT GCC ATA TGT G-3′ 5′-TGA TAA ACA ACG GAA CCT CC-3′		
*Pfdhps*	5′-TTT GTT GAA CCT AAA CGT GC-3′ 5′AAC ATT TTG ATC ATT CAT GCA AT-3′		
*Pfmdr1*	5′-TGT GTT TGG TGT AAT ATT AAA GAA CA-3′ 5′-ACA TAA AGT CAA ACG TGC ATT T-3′		Go taq (7.5 µL), F/R primer (2 µL) _nf_H_2_O (3.5 µL), gDNA (2 µL), Total volume = 25 µL
*Pfcrt*	5′-TGT CTT GGT AAA CAC GCT CA-3′ 5′-AGT TGA GAG TTT CGG ATG TT-3′		

### Appendix A.2

**Table A2:** Variants Identified from variant calling Analysis.

Chromosome	Length	Variants	Variants Rate
Pf3D7_03_v3	1,067,971	82	13,024
Pf3D7_04_v3	1,200,490	3	400,163
Pf3D7_05_v3	1,343,557	2	671,778
Pf3D7_07_v3	1,445,207	3	481,735
Pf3D7_08_v3	1,472,805	6	245,467
Pf3D7_13_v3	2,925,236	3	1,462,618
Total	9,455,266	98	96,482

## References

[1] World Health Organization WHO 2024 Malaria Report: Addressing Inequity in the Global Malaria Response. https://www.who.int/teams/global-malaria-programme/reports/world-malaria-report-2024.

[2] de Fatima Ferreira-da-Cruz M., Almeida-de-Oliveira N.K., de Abreu-Fernandes R., de Lavigne Mello A.R., Zanini G., de Abreu F.V.S., de Pina-Costa A., Lourenco-de-Oliveira R., Brasil P., Daniel-Ribeiro C.T. (2025). Simium zoonotic malaria infection in the Rio de Janeiro Atlantic forest, Brazil. Sci. Rep..

[3] Andrade M.V., Noronha K., Diniz B.P.C., Guedes G., Carvalho L.R., Silva V.A., Calazans J.A., Santos A.S., Silva D.N., Castro M.C. (2022). The economic burden of malaria: A systematic review. Malar. J..

[4] Sachs J., Malaney P. (2002). The economic and social burden of malaria. Nature.

[5] Ricci F. (2012). Social implications of malaria and their relationships with poverty. Mediterr. J. Hematol. Infect. Dis..

[6] World Health Organization World Malaria Report. https://www.who.int/teams/global-malaria-programme/reports/world-malaria-report-2023.

[7] Sarfo J.O., Amoadu M., Kordorwu P.Y., Adams A.K., Gyan T.B., Osman A.G., Asiedu I., Ansah E.W. (2023). Malaria amongst children under five in sub-Saharan Africa: A scoping review of prevalence, risk factors and preventive interventions. Eur. J. Med. Res..

[8] U.S. President’s Malaria Initiative Cameroon Malaria Profile Cameroon-Country-Profile. https://mesamalaria.org/wp-content/uploads/2025/04/CAMEROON_Malaria_Profile_PMI_FY_2024.pdf.

[9] National Malaria Control Programme Cameroon Malaria Indicators Survey. https://dhsprogram.com/pubs/pdf/PR145/PR145.pdf.

[10] World Health Organization World Malaria Report: Tracking Progress and Gaps in the Global Response to Malaria. https://www.who.int/teams/global-malaria-programme/reports/world-malaria-report-2022.

[11] El-Moamly A.A., El-Sweify M.A. (2023). Malaria vaccines: The 60-year journey of hope and final success-lessons learned and future prospects. Trop. Med. Health.

[12] The RTS,S Clinical Trials Partnership (2014). Efficacy and Safety of the RTS,S/AS01 Malaria Vaccine during 18 Months after Vaccination: A Phase 3 Randomized, Controlled Trial in Children and Young Infants at 11 African Sites. PLoS Med..

[13] Datoo M.S., Natama H.M., Somé A., Bellamy D., Traoré O., Rouamba T., Tahita M.C., Ido N.F.A., Yameogo P., Valia D. (2022). Efficacy and immunogenicity of R21/Matrix-M vaccine against clinical malaria after 2 years’ follow-up in children in Burkina Faso: A phase 1/2b randomised controlled trial. Lancet Infect. Dis..

[14] Ndoula S.T., Mboussou F., Njoh A.A., Nembot R., Baonga S.F., Njinkeu A., Biey J., Kaba M.I., Amani A., Farham B. (2024). Malaria Vaccine Introduction in Cameroon: Early Results 30 Days into Rollout. Vaccines.

[15] World Health Organization Cameroon Kicks Malaria Vaccine Rollout. https://www.afro.who.int/countries/cameroon/news/cameroon-kicks-malaria-vaccine-rollout.

[16] Lyimo B.M., Bakari C., Popkin-Hall Z.R., Giesbrecht D.J., Seth M.D., Pereus D., Shabani Z.I., Moshi R., Boniface R., Mandara C.I. (2024). Genetic polymorphism and evidence of signatures of selection in the Plasmodium falciparum circumsporozoite protein gene in Tanzanian regions with different malaria endemicity. Malar. J..

[17] Amegashie E.A., Amenga-Etego L., Adobor C., Ogoti P., Mbogo K., Amambua-Ngwa A., Ghansah A. (2020). Population genetic analysis of the Plasmodium falciparum circumsporozoite protein in two distinct ecological regions in Ghana. Malar. J..

[18] Huang H.Y., Liang X.Y., Lin L.Y., Chen J.T., Ehapo C.S., Eyi U.M., Li J., Jiang T.T., Zheng Y.Z., Zha G.C. (2020). Genetic polymorphism of Plasmodium falciparum circumsporozoite protein on Bioko Island, Equatorial Guinea and global comparative analysis. Malar. J..

[19] He Z.Q., Zhang Q.Q., Wang D., Hu Y.B., Zhou R.M., Qian D., Yang C.Y., Lu D.L., Li S.H., Liu Y. (2022). Genetic polymorphism of circumsporozoite protein of Plasmodium falciparum among Chinese migrant workers returning from Africa to Henan Province. Malar. J..

[20] Maina M., Musundi S., Kuja J., Waweru H., Kiboi D., Kanoi B.N., Gitaka J. (2024). Genetic variation of the Plasmodium falciparum circumsporozoite protein in parasite isolates from Homabay County in Kenya. Front. Parasitol..

[21] Dieng C.C., Ford C.T., Lerch A., Doniou D., Vegesna K., Janies D., Cui L., Amoah L., Afrane Y., Lo E. (2023). Genetic variations of Plasmodium falciparum circumsporozoite protein and the impact on interactions with human immunoproteins and malaria vaccine efficacy. Infect. Genet. Evol..

[22] Wicht K.J., Mok S., Fidock D.A. (2020). Molecular Mechanisms of Drug Resistance in Plasmodium falciparum Malaria. Annu. Rev. Microbiol..

[23] Lei Z.N., Wu Z.X., Dong S., Yang D.H., Zhang L., Ke Z., Zou C., Chen Z.S. (2020). Chloroquine and hydroxychloroquine in the treatment of malaria and repurposing in treating COVID-19. Pharmacol. Ther..

[24] Amambua-Ngwa A., Button-Simons K.A., Li X., Kumar S., Brenneman K.V., Ferrari M., Checkley L.A., Haile M.T., Shoue D.A., McDew-White M. (2023). Chloroquine resistance evolution in Plasmodium falciparum is mediated by the putative amino acid transporter AAT1. Nat. Microbiol..

[25] Sidhu A.B., Verdier-Pinard D., Fidock D.A. (2002). Chloroquine resistance in Plasmodium falciparum malaria parasites conferred by pfcrt mutations. Science.

[26] Miriam K.L., Christopher V.P. (2004). Withdrawing antimalarial drugs: Impact on parasite resistance and implications for malaria treatment policies, Drug Resistance Updates. Drug Resist. Updates.

[27] Sayang C., Gausseres M., Vernazza-Licht N., Malvy D., Bley D., Millet P. (2009). Treatment of malaria from monotherapy to artemisinin-based combination therapy by health professionals in urban health facilities in Yaounde, central province, Cameroon. Malar. J..

[28] Niba P.T.N., Nji A.M., Evehe M.-S., Ali I.M., Netongo P.M., Ngwafor R., Moyeh M.N., Ngum L.N., Ndum O.E., Acho F.A. (2021). Drug resistance markers within an evolving efficacy of anti-malarial drugs in Cameroon: A systematic review and meta-analysis (1998–2020). Malar. J..

[29] Lakshmanan V., Bray P.G., Verdier-Pinard D., Johnson D.J., Horrocks P., Muhle R.A., Alakpa G.E., Hughes R.H., Ward S.A., Krogstad D.J. (2005). A critical role for PfCRT K76T in Plasmodium falciparum verapamil-reversible chloroquine resistance. EMBO J..

[30] Tchuenkam P.V.K., Ngum L.N., Ali I.M., Chedjou J.P.K., Nji A.M., Netongo P.M., Ngwafor R., Niba P.T.N., Tah C.F., Nana W.D. (2024). Plasmodium falciparum dhps and dhfr markers of resistance to sulfadoxine-pyrimethamine five years (2016–2020) after the implementation of seasonal malaria chemoprevention in Cameroon. Wellcome Open Res..

[31] Deloron P., Bertin G., Briand V., Massougbodji A., Cot M. (2010). Sulfadoxine/pyrimethamine intermittent preventive treatment for malaria during pregnancy. Emerg. Infect. Dis..

[32] Amimo F., Lambert B., Magit A., Sacarlal J., Hashizume M., Shibuya K. (2020). Plasmodium falciparum resistance to sulfadoxine-pyrimethamine in Africa: A systematic analysis of national trends. BMJ Glob. Health.

[33] Guemas E., Coppee R., Menard S., du Manoir M., Nsango S., Makaba Mvumbi D., Nakoune E., Eboumbou Moukoko C.E., Bouyou Akotet M.K., Mirabeau T.Y. (2023). Evolution and spread of Plasmodium falciparum mutations associated with resistance to sulfadoxine-pyrimethamine in central Africa: A cross-sectional study. Lancet Microbe.

[34] Gatton M.L., Martin L.B., Cheng Q. (2004). Evolution of resistance to sulfadoxine-pyrimethamine in Plasmodium falciparum. Antimicrob. Agents Chemother..

[35] Flegg J.A., Metcalf C.J.E., Gharbi M., Venkatesan M., Shewchuk T., Hopkins Sibley C., Guerin P.J. (2013). Trends in antimalarial drug use in Africa. Am. J. Trop. Med. Hyg..

[36] Hassett M.R., Roepe P.D. (2019). Origin and Spread of Evolving Artemisinin-Resistant Plasmodium falciparum Malarial Parasites in Southeast Asia. Am. J. Trop. Med. Hyg..

[37] Nji A.M., Ali I.M., Moyeh M.N., Ngongang E.O., Ekollo A.M., Chedjou J.P., Ndikum V.N., Evehe M.S., Froeschl G., Heumann C. (2015). Randomized non-inferiority and safety trial of dihydroartemisin-piperaquine and artesunate-amodiaquine versus artemether-lumefantrine in the treatment of uncomplicated Plasmodium falciparum malaria in Cameroonian children. Malar. J..

[38] Assefa A., Fola A.A., Tasew G. (2024). Emergence of Plasmodium falciparum strains with artemisinin partial resistance in East Africa and the Horn of Africa: Is there a need to panic?. Malar. J..

[39] Breman J.G. (2012). Resistance to artemisinin-based combination therapy. Lancet Infect. Dis..

[40] World Health Organization Malaria: Artemisinin Partial Resistance. https://www.who.int/news-room/questions-and-answers/item/artemisinin-resistance.

[41] Fidock D.A., Rosenthal P.J. (2021). Artemisinin resistance in Africa: How urgent is the threat?. Med.

[42] Jeang B., Zhong D., Lee M.C., Atieli H., Yewhalaw D., Yan G. (2024). Molecular surveillance of Kelch 13 polymorphisms in Plasmodium falciparum isolates from Kenya and Ethiopia. Malar. J..

[43] World Health Organization Report on Antimalarial Drug Efficacy and Resistance: ACT Markers. https://www.who.int/teams/global-malaria-programme/reports/world-malaria-report-2020.

[44] Avci K.D., Karakus M., Kart Yasar K. (2024). Molecular survey of pfmdr-1, pfcrt, and pfk13 gene mutations among patients returning from Plasmodium falciparum endemic areas to Turkey. Malar. J..

[45] Manoj T.D., Alan F.C. (2005). Contribution of the pfmdr1 gene to antimalarial drug-resistance. Acta Trop..

[46] Plowe C.V. (2022). Malaria chemoprevention and drug resistance: A review of the literature and policy implications. Malar. J..

[47] Rosenthal P.J. (2018). Artemisinin Resistance Outside of Southeast Asia. Am. J. Trop. Med. Hyg..

[48] Rosenthal P.J., Asua V., Bailey J.A., Conrad M.D., Ishengoma D.S., Kamya M.R., Rasmussen C., Tadesse F.G., Uwimana A., Fidock D.A. (2024). The emergence of artemisinin partial resistance in Africa: How do we respond?. Lancet Infect. Dis..

[49] Antonio-Nkondjio C., Ndo C., Njiokou F., Bigoga J.D., Awono-Ambene P., Etang J., Ekobo A.S., Wondji C.S. (2019). Review of malaria situation in Cameroon: Technical viewpoint on challenges and prospects for disease elimination. Parasit. Vectors.

[50] Quan H., Igbasi U., Oyibo W., Omilabu S., Chen S.B., Shen H.M., Okolie C., Chen J.H., Zhou X.N. (2020). High multiple mutations of Plasmodium falciparum-resistant genotypes to sulphadoxine-pyrimethamine in Lagos, Nigeria. Infect. Dis. Poverty.

[51] Moyeh M.N., Njimoh D.L., Evehe M.S., Ali I.M., Nji A.M., Nkafu D.N., Masumbe P.N., Barbara A.T., Ndikum V.N., Mbacham W.F. (2018). Effects of Drug Policy Changes on Evolution of Molecular Markers of Plasmodium falciparum Resistance to Chloroquine, Amodiaquine, and Sulphadoxine-Pyrimethamine in the South West Region of Cameroon. Malar. Res. Treat..

[52] Takala-Harrison S., Jacob C.G., Arze C., Cummings M.P., Silva J.C., Dondorp A.M., Fukuda M.M., Hien T.T., Mayxay M., Noedl H. (2015). Independent emergence of artemisinin resistance mutations among Plasmodium falciparum in Southeast Asia. J. Infect. Dis..

[53] Liu Y., Liang X., Li J., Chen J., Huang H., Zheng Y., He J., Ehapo C.S., Eyi U.M., Yang P. (2022). Molecular Surveillance of Artemisinin-Based Combination Therapies Resistance in Plasmodium falciparum Parasites from Bioko Island, Equatorial Guinea. Microbiol. Spectr..

[54] Amusan A., Akinola O., Akano K., Hernandez-Castaneda M., Dick J.K., Sowunmi A., Hart G., Gbotosho G. (2025). Polymorphisms in Pfkelch13 domains before and after the introduction of artemisinin-based combination therapy in Southwest Nigeria. PLoS ONE.

[55] Sansonetti P.J., Lebras C., Verdier F., Charmot G., Dupont B., Lapresle C. (1985). Chloroquine-resistant Plasmodium falciparum in Cameroon. Lancet.

[56] Leonado K.B., Vincent F.N., Mathieu N., Albert S., Jean-Christian Y., Raphael T.O., Georges S. (2006). Molecular Epidemiology of Malaria in Cameroon. XXI. Baseline Therapeutic efficacy of chloroquine, amodiaquine, and sulfadoxine-pyrimethamine monotherapies in children before national drug policy change. Am. J. Trop. Med. Hyg..

[57] Lu F., Zhang M., Culleton R.L., Xu S., Tang J., Zhou H., Zhu G., Gu Y., Zhang C., Liu Y. (2017). Return of chloroquine sensitivity to Africa? Surveillance of African Plasmodium falciparum chloroquine resistance through malaria imported to China. Parasit. Vectors.

[58] Ndam N.T., Basco L.K., Ngane V.F., Ayouba A., Ngolle E.M., Deloron P., Peeters M., Tahar R. (2017). Reemergence of chloroquine-sensitive pfcrt K76 Plasmodium falciparum genotype in southeastern Cameroon. Malar. J..

[59] Moyeh M.N., Fankem S.N., Ali I.M., Sofeu D., Sandie S.M., Njimoh D.L., Ghogomu S.M., Kimbi H.K., Mbacham W.F. (2022). Current status of 4-aminoquinoline resistance markers 18 years after cessation of chloroquine use for the treatment of uncomplicated falciparum malaria in the littoral coastline region of Cameroon. Pathog. Glob. Health.

[60] Pamela C., Sandie M., Xavier I., Sandrine E.N., Majoline T.T., Luc A., Parfait H.A., Isabelle M., Antoine B. (2015). Prevalence of Plasmodium falciparum parasites resistant to sulfadoxine/pyrimethamine in pregnant women in Yaoundé, Cameroon: Emergence of highly resistant pfdhfr/pfdhps alleles. J. Antimicrob. Chemother..

[61] Ajit L., Phillipe M., Gerald V., Ansar A.P., Kent E.K., Roger B., Edwin L., Marguerite K., Magdalena P., Martine D. (1999). Potent Induction of Focused Th1-Type Cellular and Humoral Immune Responses by RTS,S/SBAS2, a Recombinant Plasmodium falciparum Malaria Vaccine. J. Infect. Dis..

[62] Ballou W.R., Voss G., Kester K.E., Heppner D.G., Krzych U. (2003). Protective immunity induced with malaria vaccine, RTS,S, is linked to Plasmodium falciparum circumsporozoite protein-specific CD4+ and CD8+ T cells producing IFN-gamma. J. Immunol..

[63] Esum M.E., Roland N.N., Sumbele I. Trends of Malaria in the South West Region of Cameroon: Overview, Challenges and Perspectives (SDG3). Proceedings of the 1st International Conference on Sustainable Development Goals "Why It Matters" As Part of the Decade of Action, Utah Valley University.

[64] Apinjoh T.O., Tata R.B., Anchang-Kimbi J.K., Chi H.F., Fon E.M., Mugri R.N., Tangoh D.A., Nyingchu R.V., Ghogomu S.M., Nkuo-Akenji T. (2015). Plasmodium falciparum merozoite surface protein 1 block 2 gene polymorphism in field isolates along the slope of mount Cameroon: A cross—Sectional study. BMC Infect. Dis..

[65] Adrien L., Tommaso L. (2019). pycoQC, interactive quality control for Oxford Nanopore Sequencing. J. Open Source Softw..

[66] Heng L. (2018). Minimap2: Pairwise alignment for nucleotide sequences. Bioinformatics.

[67] Petr D., James K.B., Jennifer L., John M., Valeriu O., Thomas K., Martin O.P., Andrew W., Shane A.M., Robert M.D. (2021). Twelve years of SAMtools and BCFtools. GigaScience.

[68] Junhao S., Zhenxian Z., Syed S.A., Tak-Wah L., Ruibang L. (2022). Clair3-trio: High-performance Nanopore long-read variant calling in family trios with trio-to-trio deep neural networks. Brief. Bioinform..

